# High sensitivity ctDNA assays in genitourinary malignancies: current evidence and future directions

**DOI:** 10.1093/oncolo/oyae198

**Published:** 2024-08-02

**Authors:** Kartik R Patel, Soroush Rais-Bahrami, Arnab Basu

**Affiliations:** Department of Urology, University of Alabama at Birmingham, Heersink School of Medicine, Birmingham, AL 35233, United States; Department of Urology, University of Alabama at Birmingham, Heersink School of Medicine, Birmingham, AL 35233, United States; Department of Radiology, University of Alabama at Birmingham, Heersink School of Medicine, Birmingham, AL 35233, United States; O’Neal Comprehensive Cancer Center, University of Alabama at Birmingham, Heersink School of Medicine, Birmingham, AL 35233, United States; O’Neal Comprehensive Cancer Center, University of Alabama at Birmingham, Heersink School of Medicine, Birmingham, AL 35233, United States; Division of Hematology-Oncology, Department of Medicine, University of Alabama at Birmingham, Heersink School of Medicine, Birmingham, AL 35233, United States

**Keywords:** circulating tumor DNA, molecular residual disease, minimal residual disease, urothelial carcinoma, renal cell carcinoma, prostate cancer

## Abstract

In the recent decade, analysis of circulating tumor DNA (ctDNA) has improved cancer care by allowing for rapid detection of actionable molecular targets. A new generation of circulating DNA tests is now becoming available commercially. These tests are characterized by a superior limit of detection of 0.01% vaF or better, allowing for the detection of radiologically occult molecular residual disease (MRD). MRD tests have the potential to revolutionize neoadjuvant and adjuvant treatment. In addition, these tests can be used as tumor markers to assess disease response. We reviewed the current evidence for the use of high-sensitivity MRD assays with particular focus on the genitourinary tumors. Multiple studies have now been reported in urothelial, renal, and recently testicular carcinoma. We find that the sensitivity varies across tumor types in the adjuvant setting and may reach a high of 100% in urothelial cancer. Specificity in tumor-informed MRD appears to be preserved across tumor types (98%-100%). Several trials are now prospectively validating MRD testing in biomarker integral studies, mainly in urothelial carcinoma.

## Teaser

Molecular residual disease (MRD) tests have the potential to revolutionize neoadjuvant and adjuvant treatment. This article reviews current evidence for the use of high-sensitivity MRD assays with particular focus on the genitourinary tumors.

**Implications for practice**

ctDNA tests with high sensitivity and specificity are now entering clinical practice. These tests can be used to detect radiologically occult disease or minimal residual disease (MRD) after surgery. Several studies appear to support the use of MRD for identifying high-risk patients in urothelial carcinoma. Emerging data suggests that these assays may provide important prognostic information in other genitourinary malignancies as well.

## The first generation of ctDNA assays

While the presence of plasma cell free DNA (cfDNA) was first noted in 1948, it took many decades to show that circulating tumor DNA (ctDNA) could be separately analyzed, and that it could be correlated with clinical course of disease.^1^ Despite this, the clinical actionability of ctDNA remained in question until the investigation of molecular drivers of cancer and the development of agents targeting these alterations. In 2003 and 2004, targeted therapies for epidermal growth factor receptor (EGFR) alterations in lung cancer, gefitinib and erlotinib were developed and ctDNA offered an easy and potentially quicker route to determine if patients were candidates for these directed therapies. This paved the way for ctDNA sampling for specific molecular alterations, with these tests aptly called the “liquid biopsy.”^2^ The Roche Cobas Test for non-small cell lung cancer became the first US Food & Drug Administration approved plasma ctDNA liquid biopsy to evaluate EGFR alterations in 2016. In subsequent decades, treatments have been developed toward other targets such as BRCA1/BRCA2, ATM and KRAS, and genomic alterations such as TP53, RB, or PTEN, have been shown to have important prognostic implications for cancers. Expansion of these actionable alterations have supported the development and commercialization of tests, such as Guardant 360 (Guardant Health Inc.) or Foundation One Liquid CDX (Roche, Inc.), that look for a broad panel of clinically relevant alterations. These tests use next generation sequencing technology with multiplexed assays directed toward prespecified targets.^3^ Their primary purpose is to provide a relatively quick and reliable evaluation of the presence or absence of these actionable mutations. These tests are designed to be used in a metastatic setting and thus are not best suited to the detection of cancer that may be occult.

## High-sensitivity ctDNA assays

Current methods of assessing treatment efficacy and disease progression rely on conventional imaging, such as computed tomography and magnetic resonance imaging, and in some cases, tumor markers. An occult 1 gram tumor may contain up to 1 × 10^8^ cells, all of which are connected to the circulation due to the need for oxygen and nutrients.^4^ Thus, there is potential for the detection of these radiologically occult tumors through ctDNA analysis. The first generation ctDNA tests described previously are not ideally suited for use as tumor markers in this low volume state. A new generation of high sensitivity and specificity ctDNA assays have been developed for solid tumors termed molecular residual disease (MRD) tests that may achieve detection of micrometastases. MRD tests require deep limits of detection with analytical sensitivity of more than 98% at ctDNA VAF of 0.01%-0.02% in conjunction with a high specificity exceeding 95% to enable the detection of occult disease.^5^ These tests may be either tumor informed or tumor agnostic. Tests such as Signatera (Natera Inc.) and Personalized Cancer Monitoring (Invitae Corp.) use whole exome sequencing, and tests like Precise MRD (Myriad Genetics) use whole-genome sequencing of the original tumor to develop a personalized mutation profile, which is then used to create a patient-specific probe. Other tests such as Guardant Reveal (Guardant health Inc.) utilize predetermined somatic alterations, as well as validated epigenomic cancer-specific methylation signatures, to identify the presence of cancerous DNA with respect to normal DNA and offers the advantage of a quicker turnaround without having a need for biopsy.^6^ Early clinical data on the application of MRD based ctDNA has been promising, and we review the current evidence for their use in the genitourinary malignancies.

## ctDNA in urothelial carcinoma

Urothelial carcinoma (UC) is known to have a high rate of DNA shedding.^7^ Early studies of ctDNA in bladder cancer have established that high plasma and urine ctDNA levels demonstrate an association with disease progression and metastatic disease.^8-11^ The performance of MRD testing was first reported using a tumor-informed ctDNA in a prospective cohort of muscle invasive urothelial cancer patients who were followed during all therapy.^12^ In this study of 68 patients, a bespoke assay using Natera’s Signatera was developed using tumor-specific mutations identified through SeqCap whole exome sequencing of the tumor and germline cells. ctDNA levels were analyzed at various clinically important checkpoints, including before neoadjuvant chemotherapy, after neoadjuvant chemotherapy but before cystectomy, and at serial intervals after cystectomy. The median follow-up at the time of report was 21 months after cystectomy. MRD positivity identified all patients who had eventual relapse during this follow-up period with a median lead time of 96 days. MRD positivity prior to neoadjuvant chemotherapy was strongly prognostic. 46% (11 of 24) of these patients recurred during reported follow-up, with 10 of these 11 patients recurring within 12-months. Only 3% (1 of 35) of patients who had MRD negativity prior to neoadjuvant chemotherapy demonstrated eventual recurrence.^12^

The second time point (after chemotherapy and before cystectomy) also showed significant prognostic ability as there was a 75% (6 of 8 patients) overall and 12-month recurrence rate among ctDNA-positive patients. The recurrence rate for ctDNA negative patients was 11% (6 of 55 patients) at all follow up and the 12-month recurrence rate was 7% (4 of 55 patients). 100% of patients who had ctDNA detection before cystectomy demonstrated residual tumor (stage ≥ T1) at time of cystectomy. Lastly, the third checkpoint (after cystectomy) demonstrated the most significant results as the overall and 12-month recurrence rate in ctDNA positive patients was 76% (13 of 17 patients) and 59% (10 of 17 patients), respectively. Of great importance, the overall 12-month recurrence rate was 0% for ctDNA negative patients. Overall MRD testing showed a 100% (13 of 13 patients) sensitivity and 98% (48 of 49 patients) specificity for detecting post-cystectomy recurrent disease^12^

Furthermore, supportive evidence for MRD in urothelial carcinoma comes from the analysis of the ImVigor010 trial of adjuvant atezolizumab in patients with high-risk resected urothelial carcinoma. Overall, this trial failed to show a benefit of adjuvant atezolizumab in high-risk resected patients. However, serial MRD testing, using the previously described bespoke ctDNA test (Signatera, Natera Inc.), was conducted in a large subset of patients, herein called the biomarker evaluable population (581 of 809 patients). In the prespecified exploratory analysis of these patients, at cycle 1 day 1 (C1D1) of therapy, 37% (241 of 581) of patients in the study were ctDNA positive. These patients had improved disease-free survival when treated with adjuvant atezolizumab compared to those that did not receive adjuvant treatment HR 0.58 (95% CI: 0.43-0.79). When considering ctDNA samples from C1D1 and cycle 3 day 1 (C3D1), 38.4% (186 of 485) of patients were ctDNA positive at C3D1, and these patients had a greater risk of disease progression and relapse than ctDNA negative patients. More importantly, patients who cleared their ctDNA levels (ctDNA positive at C1D1 but ctDNA negative at C3D1) after treatment with atezolizumab showed improved overall survival in this exploratory analysis (OS HR = 0.14 (95% CI: 0.03-0.59).^13^

A ctDNA-based analysis was also conducted in the ABACUS trial, which studied 2 doses of neoadjuvant atezolizumab prior to cystectomy for muscle invasive urothelial carcinoma. ctDNA monitoring was performed using Signatera, Natera Inc. before and after neoadjuvant therapy. In this trial, 62.5% (25 of 40) of patients had positive ctDNA prior to radical cystectomy. Of the samples available after neoadjuvant therapy (17 patients), 18% (3 of 17) patients had ctDNA clearance. Patients that did not respond to treatment had no changes in ctDNA levels.^14^ Young et al examined 2 different methods of determining ctDNA response in another analysis of the ABACUS study: ctDNA clearance and 50% reduction in ctDNA variant allele frequency (VAF). Using these 2 methods, patients that were defined with ctDNA clearance showed pathologic complete response (pCR) to treatment without relapse while a 50% reduction threshold did not appear accurate in prediction of response. Despite this, the study showed a possible use of ctDNA kinetics, defined as MRD clearance, to inform neoadjuvant therapy.^15^

Recently, tumor-informed MRD (Signatera, Natera Inc.) was examined in a retrospective real-world cohort of 109 patients with muscle-invasive bladder cancer, at a median follow-up of 13.6 months, ctDNA positivity was associated with shorter disease-free survival (DFS HR = 9.9, (95%CI: 4.1-24). During follow-up, 97% of patients who tested negative for MRD did not show any evidence of recurrent illness, while 63% (29 of 46) patients who were MRD positive had recurred by the time of report. ctDNA identified recurrence with a mean lead time of 61 days.^16^ In a follow-up analysis of this expanded cohort, 46 patients with pure or mixed variant histology bladder tumors were also examined for MRD using the same assay. ctDNA positivity during the surveillance period after cystectomy was strongly associated with recurrence risk (DFS HR = 55.26, (95%CI: 5.3-7648, *P* < .00001) while none of the serially negative patients had a recurrence.^17^

Carrasco et al used a tumor-agnostic approach looking at ctDNA dynamics as a biomarker for muscle-invasive bladder cancer after radical cystectomy and at various time points following surgery. Specific alterations in the *TERT* and *ATM* genes were interrogated in ctDNA using digital droplet PCR (ddPCR). Results from this study showed that 46% (17 of 37) of patients had progressed during a median follow-up of 36 months. Using multivariate Cox regression analysis, ctDNA levels 4 months after radical cystectomy was shown to be an independent prognostic biomarker for disease progression while positive ctDNA status was an independent biomarker for cancer specific survival.^18^

In a neoadjuvant study testing the use of ipilimumab and of nivolumab before surgery for UC (Phase 1B NABUCCO trial), patients in Cohort 1 of the trial were treated with neoadjuvant ipilimumab and nivolumab.^19-21^ Using the RaDar (Neogenomics Laboratories Inc.) ctDNA detection assay, plasma ctDNA was undetectable in 93% (13 of 14) patients who had a response to therapy defined as ypT0/Tis/Ta/T1N0 for biomarker purposes vs 40% of non-responders (4 of 10) (Table 1).^21^

**Table 1.** Summary of urothelial carcinoma clinical studies with high-sensitivity plasma ctDNA.

Author	Year	Assay	Setting	N	Sensitivity	Specificity	PFS hazard ratio	Lead time	Reference
Christensen	2019	Bespoke PCR (Signatera, Natera Inc.)	MIBC	68	100%	98%	129.6	13.7 weeks	Christenson^12^
Powles	2021	Bespoke PCR (Signatera, Natera Inc.)	MIBC	581	59%	99.8%	0.58	NR	Powles^13^
Young	2024	Bespoke PCR (Signatera, Natera Inc.)	MIBC	40	NR	NR	NR	NR	Young^15^
Kommalapati	2023	Bespoke PCR (Signatera, Natera Inc.)	MIBC	109	94%	NR	9.9	61 days	Kommalapati^16^
Carrasco	2022	Agnostic ddPCR	MIBC	37	NR	NR	4.199	NR	Carrasco^18^
van Dorp	2023	Bespoke PCR (RaDaR)	MIBC	17	NR	100%	10.4	NR	van Dorp, 2023^21^

Abbreviations: MIBC, muscle-invasive bladder cancer, NR, not reported.

While a significant amount of literature has focused on plasma ctDNA, recent studies have also investigated urinary tumor DNA (utDNA) secondary to bladder cancer cells shedding tumor DNA directly into urine. In a study of 74 patients who underwent either neoadjuvant therapy or radical cystectomy, utDNA analyzed by uCAPP-seq and ultra-low-pass whole-genome sequencing (ULP-WGS) was found to have 87% sensitivity in predicting residual disease. Progression-free survival (PFS) and OS were both worse for patients who had MRD positivity.^22^ This study shows that urine cfDNA can also be used in detecting MRD in patients with bladder cancer as an adjunct to plasma ctDNA. Similarly, Zhang et al conducted a study of 20 patients treated with 2-4 cycles of toripalimab every 2 weeks before receiving a radical cystectomy and used the PredicineBEACON MRD detection assay. Neoadjuvant toripalimab therapy led to 8 out of 20 patients (40%) obtaining pCR, and of these 8 patients, 100% had utDNA MRD responses that matched pCR.^23^

A study by Christensen et al looked at 92 patients undergoing cystectomy for urothelial carcinoma with a tumor-informed 50-gene PCR panel, in addition to ctDNA from plasma, utDNA from urine supernatant, and urinary cell pellets were also analyzed.^24^ Prior to a cystectomy, 89% of urine supernatants, 85% of urine pellets, and 43% of plasma samples tested positive for illness. Plasma ctDNA correlated with a lack of response to neoadjuvant therapy. Urine supernatants displayed a noteworthy association with PFS and demonstrated a 0% recurrence rate for patients who had cleared utDNA. Combining plasma samples and urine supernatant DNA dynamics, there was 71% (17 of 24 patients) concordance with clinical outcomes.^24^ However, there remains variability based on the assay methods. In a recent study, van Dorp et al found that the absence of utDNA showed no correlation to pathological response in muscle-invasive urothelial carcinoma in the NABUCCO trial, while clearance of plasma ctDNA did (Table 2).^21^

**Table 2.** Summary of urothelial carcinoma clinical studies in urinary cfDNA

Author	Year	Assay	Setting	N	Sensitivity (%)	Specificity	PFS hazard ratio	Lead time	Reference
Chauhan	2023	Bespoke PCR (uCAPP-SeQ + ULP-WGS)	MIBC	74	87	NR	3.00	NR	Chauhan^22^
Zhang	2023	Bespoke PCR (PredicineBEACON)	MIBC	20	100	99%	NR	NR	Zhang^23^
Christensen	2023	Bespoke 50-gene PCR	MIBC	92	72	65%	NR	NR	Christensen^24^

Abbreviations: CAPP-Seq, CAncer personalized profiling by deep sequencing; MIBC, muscle-invasive bladder cancer; MRD, molecular residual disease; NAC, neoadjuvant chemotherapy; NR, not reported; WES, whole exome sequencing; WGS, whole-genome sequencing.

## ctDNA in renal cell carcinoma

The use of ctDNA is still immature in metastatic RCC (mRCC). In an early study by Kim et al, a positive correlation between ctDNA status and tumor burden was observed.^25^ ctDNA levels decreased after immune checkpoint inhibitor therapy, importantly, ctDNA status and fragment size demonstrated a significant association with PFS and cancer specific survival. Overall, there was greater ctDNA detection in mRCC in comparison to localized RCC.^25^ Among studies of ultrasensitive ctDNA, Chehrazi-Raffle et al tested a tumor-informed approach to ctDNA using targeted digital sequencing [TARDIS] in 12 patients with mRCC undergoing systemic therapy.^26^ Of these 12 patients, those with a CR appeared to have lower ctDNA concentrations when compared to patients with those with a partial response (PR). Median post-treatment ctDNA in patients with a CR was 0.007% compared to a median ctDNA of 0.181% in patients with a PR. Furthermore, 6 patients had radiographic progression, and these 6 patients had ctDNA levels significantly higher than the other 6 patients that did not progress. Patients who had progressed had median ctDNA levels of 0.362% and those that maintained their response had a median ctDNA level of 0.033%. This approach of personalized ctDNA analysis using TARDIS provided high sensitivity and specificity in mRCC.^26^ In another study of 21 patients with mRCC treated with sunitinib, Peterson et al showed promising results using Signatera.^27^ Among the patients, 17 out of 21 (81%) had positive ctDNA tests.. Of these 17, after 6 weeks of sunitinib treatment, 2 (12%) had ctDNA clearance, 4 (24%) had decreased ctDNA levels, and of interest, 10 (59%) showed increased ctDNA. Ten patients showed disease progression based on imaging, 8 (80%) of which showed increased levels of ctDNA after the 6-week treatment with sunitinib.^27^ A recent study of 41 patients with RCC treated in a real-world setting used the same tumor informed assay (Signatera, Natera Inc.) to evaluate ctDNA kinetics in high risk resected or metastatic RCC treated with immunotherapy. ctDNA detection at any timepoint was 70%, with a median follow-up of 26 weeks at the time of reporting. Fourteen patients in the cohort experienced disease progression. All 14 patients had detectable ctDNA before radiological progression. Additionally, there was a 78% concordance rate (32 of 41 patients) between clinical outcome and ctDNA status.^28^ Finally, Jang et al performed a study of 18 patients with various advanced GU malignancies, predominantly mRCC (75% renal cell, 17% urothelial, and 8% prostate). This study had a detection rate of 50%, and there was a concordance rate of 83% (15 of 18 patients) between ctDNA detection and restaging imaging. Two of 3 discordant progressors had CNS progression.^29^

Studies have also used tumor-informed ctDNA for MRD detection in localized RCC. In a retrospective study of 42 patients on a cohort of localized renal cell carcinoma patients who had undergone nephrectomy, 14 of 34 (41%) patients had ctDNA detected at baseline. After surgery, 100% (8 out of 8) of the ctDNA positive patients experienced a relapse. However, 48% (16 of 33) of patients who were ctDNA negative relapsed after surgery (NPV = 52%).^30^ Recently this assay was validated in a prospective study of 82 patients with localized renal cell carcinoma. Of the 82 cases, 76% (62) of patients were ctDNA negative. Of these 62 ctDNA patients, 92% (57 of 62) of patients had no evidence of disease or disease that did not progress during reported follow-up. Among the patients that had positive ctDNA (20 of 82), 65% (13 of 20) demonstrated concordance between radiographic imaging and ctDNA status.^31^ As with previous studies, a positive MRD indicated an extremely high risk of progression, with all patients eventually experiencing relapse during reported follow-up (Table 3).

**Table 3.** Summary of renal cell cancer carcinoma studies.

Author	Year	Assay	Setting	N	Sensitivity	Specificity	PFS hazard ratio	Lead time	Concordance rate	Reference
Kim	2021	Bespoke PCR	Localized RCC & mRCC	20	NR	NR	NR	NR	NR	Kim, 2021^25^
Chehrazi-Raffle	2023	Bespoke PCR (TARDIS)	mRCC	12	NR	NR	NR	NR	60%	Chehrazi-Raffle^26^
Peterson	2022	Bespoke PCR (Signatera, Natera Inc.)	mRCC	21	81%	NR	4.9	NR	NR	Peterson^27^
Basu	2023	Bespoke PCR (Signatera, Natera Inc.)	High risk resected or mRCC	41	71%	NR	NR	13.6 weeks	78%	Basu^28^
Jang	2023	Bespoke PCR (Signatera, Natera Inc.)	mRCC, urothelial, & prostate	18	50%(for RCC)	NR	NR	13.6 weeks	83%	Jang, 2023^29^
Correa	2019	Bespoke PCR (Signatera, Natera Inc.)	Localized RCC	42	NR	100%	2.8	NR	NR	Correa^30^
Smigelski	2023	Bespoke PCR (Signatera, Natera Inc.)	Localized RCC	82	NR	NR	NR	NR	65%	Smigelski^31^

Abbreviations: RCC, renal cell carcinoma; mRCC, metastatic renal cell carcinoma; MRD, molecular residual disease; NR, not reported; TARDIS, targeted digital sequencing.

## ctDNA in other genitourinary cancers

While there has been considerable work on circulating tumor cells and other circulating biomarkers, ctDNA-based biomarkers have been underexplored in prostate cancer. There are various challenges that arise from using liquid biopsy. Prostate cancer tends to be less molecularly diverse, as well as associated with relatively low levels of circulating tumor fragments.^32^ There is limited data on modern high-sensitivity MRD assays in prostate cancer, and traditional assays tend to perform poorly. For example, in a study with 112 localized patients with prostate cancer, ctDNA could not be detected regardless of PSA level nor tumor aggressiveness.^33^ Although detectability does increase with increasing tumor burden, in a study of 7 patients with metastatic prostate cancer, only 4 patients had detectable levels of ctDNA. Limitations in plasma ctDNA testing for prostate cancer revolve around finding the proper balance of high specificity and sensitivity in the setting of low ctDNA content and lack of unique molecular features of each tumor.^34^ The presence of ctDNA in prostate cancer thus usually portends a worse prognosis. Lau et al identified localized prostate cancer in 2 of 8 patients using personalized WGS. The detection of ctDNA was also associated with rapid progression to metastatic disease.^35^ Despite difficulties in localized prostate cancer, recent studies are adding to the body of knowledge regarding plasma ctDNA use in metastatic prostate cancer. Data from high-sensitivity MRD assays are awaited in prostate cancer.

Finally, high-sensitivity ctDNA may inform clinical decision-making for germ cell tumors, particularly for higher staged disease. A large majority of germ cell tumors are diagnosed as an isolated testicular mass managed surgically with radical orchiectomy (stage I). Post-orchiectomy surveillance is of great importance, in particular for non-seminomatous germ cell tumors with the presence of high-risk pathological factors such as lymphovascular invasion, and according to some experts, a significant proportion of embryonal histology. These patients may have a retroperitoneal recurrence risk up to 50%.^36^ Consensus guidelines recommend active surveillance vs adjuvant chemotherapy, which in these patients who are frequently young, may lead to lifelong toxicities. Additionally, patients with more advanced disease may also undergo primary definitive or salvage surgical management via retroperitoneal lymph node dissection, often with different surgical templated boundaries depending on the timing and extent of disease being addressed. In these patients, identification of true eradication of disease is critical. ctDNA has a potential role across all these stages of disease. Some early studies have now prospectively evaluated the performance of tumor-informed ctDNA in these clinical settings. The following studies have all used the tumor-informed commercially available Signatera assay.

In one study of 35 patients with testicular cancer stages I-III, including both seminomas and non-seminomas. Pre-orchiectomy ctDNA was detected in 91.6% (11 of 12) of stage I patients, and 100% of stage II or III (3 of 3) patients. During MRD-based surveillance, patients with ctDNA positivity at any time had an overall lower EFS (HR 11.8, (95% CI: 2.3-59.1)).^37^ In another study of 25 patients with stage II (28%; 7 of 25) and stage III (72%; 18 of 25) non-seminomatous germ cell tumors, Hassoun and others demonstrated a 0% recurrence rate (0 of 6) among patients who tested ctDNA negative after definitive surgery or chemotherapy. These findings were replicated in the refractory setting as patients who turned ctDNA negative post-therapy showed no further events of progression. The presence of ctDNA correlated with a reduced EFS in this study (HR = 12.98, 95%CI 1.17-1869).^38^ These 2 early studies provide valuable initial insights about the potential for high-sensitivity ctDNA analysis in better treating and predicting recurrence for germ cell tumors.

## Discussion

We describe here a selection of studies that span the different genitourinary malignancies, including localized and advanced disease. Despite the variations across studies, it is clear that high-sensitivity ctDNA MRD assays can serve as a valuable complement to radiological assessment in determining residual disease and prognostication regarding outcomes. Currently, the strongest potential application of MRD assessment among genitourinary tumors appears to be for adjuvant therapy selection in urothelial carcinoma. Despite the topline failure of ImVigor10, a strong trend was noted in the exploratory analysis for adjuvant therapy in MRD-positive patients. Given these findings, a prospective trial, ImVigor011 is currently accruing, where patients with high risk resected urothelial carcinoma will be randomized to treatment with atezolizumab versus observation only on developing MRD positivity [NCT02302807]. Also, the nonrandomized TOMBOLA trial [NCT04138628] is testing a similar design. The recently activated MODERN trial [NCT05987241] will prospectively test both an escalation in MRD-positive patients with the addition of LAG-3 inhibitor relatlimab to nivolumab, as well as de-escalation in MRD negative patients with observation only, with option to administer nivolumab upon MRD positivity, compared to a nivolumab all comer strategy. Other smaller escalation trials are also being planned with novel agents in this space. In renal cell carcinoma, the ongoing MRD guided adjuvant therapy in RCC (MRDGATE RCC) [NCT06005818] trial will enroll 100 patients and assign them to treatment with pembrolizumab vs observation based on MRD status during a screening window to provide initial estimates of DFS and OS. While many of these studies utilize tumor-informed high-sensitivity ctDNA, newer techniques are being developed that may improve limits of detection (LOD) further, such as whole-genome sequencing (Precise MRD, Myriad Genetics). Improvements in tumor agnostic assays also continue to occur such as with the use of cfMeDIP-sequencing (Adela Bio), which allows for high-sensitivity DNA methylome profiling to detect MRD. Initial data from these approaches appear very promising even in relatively low shedding tumors such as renal cell carcinoma^39,40^ Prospective studies such as the ORACLE study [NCT05059444] will provide prospective validation of tumor agnostic MRD assays in multiple cancers including the genitourinary malignancies. In summary, high-sensitivity MRD assays appear to be closely related to clinical outcomes in urothelial carcinoma, germ cell tumors, and renal cell carcinoma. These assays can be used to detect MRD in plasma but also in biofluids such as urine, with excellent sensitivity. In the future, the characteristics of high-sensitivity ctDNA analysis in each tumor type need to be validated prospectively but hold much promise for treatment assignment, escalation, and de-escalation across genitourinary malignancies.

## Conclusion

Currently, clinicians rely on pathologic staging as the primary surrogate for the likelihood of recurrence. This is specially relevant in the genitourinary malignancies where adjuvant therapy is indicated, such as in urothelial carcinoma. Emerging evidence suggests that novel ultrasensitive ctDNA assays may detect an overwhelming majority of patients with post-resection residual disease in urothelial carcinoma, especially with longitudinal monitoring. This has great implications for adjuvant therapy, and several clinical trials to prospectively validate MRD-based adjuvant therapy in urothelial carcinoma are ongoing. High-sensitivity ctDNA in other genitourinary malignancies such as testicular and renal cell carcinoma may also provide valuable prognostic information. More prospective studies are needed to validate ultrasensitive ctDNA-directed treatment approaches in these genitourinary malignancies.

## Data Availability

No new data were generated or analyzed in support of this research.
